# The frequency of ABO blood group maternal–fetal incompatibility, maternal iso-agglutinins, and immune agglutinins quantitation in Osogbo, Osun State, South-West of Nigeria

**DOI:** 10.4103/0973-6247.75998

**Published:** 2011-01

**Authors:** Bashiru S. Oseni, Oluseun F. Akomolafe

**Affiliations:** *Department of Biomedical Sciences, College of Health Sciences, Ladoke Akintola University of Technology, P.M.B. - 4000, Ogbomosho, Oyo State, Nigeria*

**Keywords:** ABO blood groups, frequency, maternal–fetal incompatibility

## Abstract

**Background:**

ABO incompatibility in maternal–fetal relationship has been shown to cause hemolytic disease of the newborn (HDNB); a survey which is not yet done in this locality.

**Aim:**

Frequency of ABO blood group maternal-fetal incompatibility, maternal iso-agglutinins, and immune agglutinins quantitation was carried out in Osogbo, Osun State, South-West of Nigeria.

**Settings and Designs:**

A total of 260 subjects comprising 130 postpartum mothers within the age range of 22-35 years having good obstetrics history and normal delivery, with their 130 neonate babies were used for the study.

**Materials and Methods:**

ABO cell and serum groupings were carried out on the subjects using standard antisera and cells with appropriate controls. Direct Coomb’s Test was carried out on neonate red cells. Antibody quantitation by double dilution on the maternal serum using red cells containing corresponding antigen to the antibody was determined. A titer, which is the reciprocal of the highest dilution showing agglutination by Indirect Coombs Test, was determined. Another batch of sera was pretreated with 2-mecarptoethanol before determining the titer.

**Statistical Analysis:**

The distribution study results obtained were compared in percentages, whereas the antibodies quantitation was expressed as titers using the mode of the titers for compariso-agglutininsn.

**Results and Conclusions:**

Thirty-eight percent (50) mothers were ABO incompatible with their babies, whereas 62% (80) mothers were compatible. The distribution of blood groups in the compatible population showed blood group O (45%); A (30%); B (20%); and AB (5%). Mothers O, A, and B carrying incompatible babies had a frequency of 24% each, whereas mothers AB had 28%. Serologist differences occur in maternal ABO antibodies of corresponding incompatible baby ABO antigens. A high incidence of ABO maternal-fetal incompatibility observed without detection of immune agglutinins is indicative of a rare incidence of HDNB due to ABO incompatibility in the population studied.

## Introduction

The ABO blood group system was discovered by Karl Landsteiner, Decastello, and Sturli.[1 2] Its inheritance described by Bernstein in 1924[3] occur from both parents through allelomorphic genes A, B, O resulting in different phenotypes A, B, AB, and O.[4 5] The fetus may inherit father’s group whose antigen on red blood cell has corresponding antibodies in the mother resulting in maternal–fetal ABO incompatibility.[6] Anti-A and anti-B are usually naturally occurring IgM[7] with immune forms produced by either transfusion or pregnancy.[8] Problems envisaged from maternal plasma antibodies in close proximity to the fetal red cell corresponding antigen stimulated this study, which is aimed at assessing the following:

- frequency of ABO maternal–fetal incompatibility yet to be surveyed in this environment;
- maternal serologic response; and
- its effect on fetal red cells at normal delivery of a normal pregnancy.

## Materials and Methods

The study, after obtaining ethical approval from the ethical committee of Ladoke Akintola University of Technology Teaching Hospital Management Board and the Health Management Board of the Ministry of Health of Osun State, was conducted at the labor wards of Ladoke Akintola University of Technology Teaching Hospital, the primary Health Centre Atelewo, and Our Lady of Fatima Catholic Hospital, Jaleyemi, all in Osogbo metropolis, South-Western Nigeria, between January 2009 and June 2009.

A total of 130 subjects within the age range of 22–35 years who had good obstetrics history and normal delivery of a baby each were recruited for the study. Exclusion criteria for the study included women with the following health problems:

- High blood pressure.
- Metabolic diso-agglutininsrders, such as diabetics.

Collection of blood samples from the mother and the baby was done with the expertise of a Consultant Obstetrician. Whole blood from the mother (5.0 mL) and the baby (2.0 mL) were collected into separate labeled dry, plain, clean bottles and allowed to clot. After 1 h standing at room temperature for complete clot retraction, the sera samples were separated after centrifugation at 5000 rpm for 10 min. The sera samples were stored at –20°C, while the red cells were stored at 4°C until the time of analysis.

Direct Coomb’s Test (DCT) was done on the babies’ cells according to the method of Knowles, 2001.[9] ABO cells and serum grouping were done on the mothers’ and the babies’ samples according to the methods of Knowles.[10] Antibodies detection and quantitation were done on the mothers’ serum samples according to the method of Knowles[11] and Regan *et al*. 2001.[12] Standard cells containing applicable antigens were used to titrate serially double diluted serum of the mother carrying incompatible baby as test and mother carrying compatible baby as control. Titer is defined as the reciprocal of the highest dilution that shows agglutination using Indirect Coomb’s Test according to the method of Knowles, 2001.[9,11] The mode of titers obtained for each control and test samples were recorded as shown by Table 1. For differentiation of IgM from IgG antibodies, the sera samples of both test and control were pretreated with 2-mercaptoethanol according to the method of Regan *et al*., 2001,[12] after which the titration was repeated as done with the untreated sera samples.

**Table 1:** The distribution of blood groups in the compatible population

Blood group		Percentage
Mother	Baby	
O	O	45
A	A	30
B	B	20
AB	AB	5

## Results

Of the 130 subjects under study, 38% (50) presented with ABO incompatibility, whereas 62% (80) were ABO compatible.

Distribution of the blood groups in the incompatible and compatible populations was shown in Tables 2 and 3, respectively. The incompatible population revealed equal distributions [24% (12)] in babies of mothers O, A, and B with a slightly higher distribution [28% (14)] in babies of mothers AB. In the compatible population, blood group O was most prominent [45% (36)], followed by group A [30% (24)], then group B [20% (16)] with group AB [5% (4)] as the least. DCT done on all the red cells of the babies were negative.

**Table 2:** The distribution of blood groups in the incompatible population

Blood group		% Frequency
Mother	Baby	
O	A	16
	B	8
A	O	8
	B	4
	AB	12
B	O	12
	A	4
	AB	8
AB	A	12
	A	12
	B	16

**Table 3:** Antibody quantitation results

Test	Titer mode	Control	Titer mode
Mother O	Anti-A 128	Mother O	Anti-A 64
Baby A	Anti-B 64	Baby O	Anti-B 128
Mother O	Anti-A 64	Mother O	Anti-A 64
Baby B	Anti-B 128	Baby O	Anti-B 128
Mother A	Anti-B 128	Mother A	Anti-B 64
Baby B		Baby A	
Mother B	Anti-A 64	Mother B	Anti-A 64
Baby A		Baby b	

Table 1 shows the results expressed as mode obtained from the titers of test and control groups. A double-fold increase in the mode titer of anti-A antibodies (128) of group O mothers carrying group A babies when compared with the control (64) was observed. Similarly, there was a double-fold increase in the mode titer of anti-B antibodies (128) of group O mothers carrying group B babies against control (64). Also, mothers A carrying babies B showed a double-fold rise in the mode titer of anti-B (128) compared with control (64), whereas the mode titer of anti-A (64) in mother B carrying baby A remained as the compatible mother B (64) carrying baby B. Negligible to no agglutination was observed in all the 2-mercaptoethanol pretreated sera.

## Discussion

ABO incompatibility in maternal–fetal relationship has been shown to cause Hemolytic Disease of the Newborn (HDNB).[13]

ABO incompatibility frequency of 38% compared with gestation compatibility of 62% showed a wide distribution difference in the 2 groups of population. This study showed almost a double-fold (38%) incompatibility frequency when compared with Caucasian populations, which showed about one fifth of all pregnancies (20%) having ABO incompatibility between fetus and mother.[14]

The distribution of the blood groups in the compatible population is in line with the frequency of ABO distribution observed in Caucasians and Africans.[6 15] The incompatible gestation showing equal distribution (24%) of babies from mothers O, A, and B with higher distribution of babies from mothers AB (28%) shows a reflection of the influence of inheritance of dominant genes A and B by the mother as earlier documented by Calafell and Francesc, 2008.[16]

Negative DCT result from all the babies’ red cells is in consonance with the 2-mercaptoethanol pretreatment of mothers’ sera result indicating the antibodies present to be of IgM type, which had been confirmed not to cross the placenta.[17]

A double-fold increase in the IgM antibodies of mothers corresponding to the fetal ABO antigen except group B mothers can be explained by soluble ABH glycoprotein substances from the fetal circulation[18] entering the mother’s circulation by diffusion, causing antigenic stimulation of IgM naturally occurring antibodies production in the maternal circulation.

The study has shown that the incidence of HDNB due to ABO gestational immunization is very rare in this community at normal pregnancy term and delivery.

## References

[1] Ali N, Anwar M, Bhalti FA, Naddem M, Ali M (2005). Frequency of ABO and Rh blood groups n major ethnic groups and casts of Pakistan. Pakistan J. Med. Sci.

[2] Sealey RR, Stephene TD, Tate P (1998). Anatomy and Physiology.

[3] Crow J (1993). Felix Bernstein and the first human marker locus. Genetics.

[4] Yazer M, Olsson M, Palcic M (2006). The Cis-AB blood group phenotype: fundamental lesions in glycobiology. Transfus Med Rev.

[5] Y Oka, N Nilkawa, A Yoshida, H Matsumoto (1982). An unusual case of blood group ABO Inheritance: O from AB X O. Am J Hum Genet.

[6] Ogasawara K, Bannai M, Saitou N (1996). Extensive polymorphism of ABO blood group gene: three major lineages of the alleles for the common ABO phenotypes. Hum Genet.

[7] Schwarting R, Wlliam DK, Steven McKenzie, Mohammad Alomari, Rubin E, Gorstein F, Rubin R, Schwarting R, Strayer D (2005). Haematopathology. Rubins Pathology: Clinicopathologic Foundations of Medicine.

[8] Contreras M, Lubenko A, Hoffbrand AV, Lewis SM (1989). Antigens in Human Blood. Postgraduate Haematology.

[9] Knowles SM, Lewis SM, Bain BJ, Bates I (2001). Laboratory aspects of blood transfusion In. Dacie and Lewis: Practical Haematology.

[10] Knowles SM, Lewis SM, Bain BJ, Bates I (2001). Laboratory aspects of blood transfusion In. Dacie and Lewis: Practical Haematology.

[11] Knowles SM, Lewis SM, Bain BJ, Bates I (2001). Red cell antigens and antibodies: erythrocytes, platelets and granulocytes In. Dacie and Lewis: Practical Haematology.

[12] Regan F, Newlands M, Barbara JM, Lewis SM, Bain BJ, Bates I (2001). Acquired haemolytic anaemias.

[13] Geifman-Holtzman O, Wojtowycz M, Kosmas E, Artal R (1997). Female allo-immunization with antibodies known to cause haemolytic disease. Obstet Gynaecol.

[14] http//www.obgyn.net/english/pubs/features/presentations/panda/3/ABO-Rh.ppt.

[15] Seltsam A, Hallensleben M, Kollman A, Blasczyk R (2003). The nature of diversity and diversification at the ABO locus. Blood.

[16] Calafell F, Roubinet F, Ramirez-Soriano A, Saitou N, Bortranpetit J, Blancher A (2008). Evolutionary dynamics of the man ABO gene. Hum Genet.

[17] Molliso-agglutininsn PL, Engelfriet CP, Contres M (1997). Blood Transfusion in Clinical Medicine. Blackwell Science.

[18] Szulman AE (1964). The histological distribution of the blood group substances in man as disclosed by Immunofluorescence III- The A,B and H antigens in embryos and fetuses from 18mm in length. J Exp Med.

